# Gastric Carcinomas and Point-of-Care Ultrasound (POCUS): A Report of Two Cases

**DOI:** 10.7759/cureus.73869

**Published:** 2024-11-17

**Authors:** Vui H Chong

**Affiliations:** 1 Department of Medicine, RIPAS Hospital, Bandar Seri Begawan, BRN

**Keywords:** gastric neoplasm, point-of-care-ultrasound, stomach cancer, trans-abdominal ultrasound, ultrasound diagnosis

## Abstract

Stomach cancer remains a significant cause of mortality, as most patients are diagnosed at advanced stages. The primary method for diagnosis is endoscopy, along with tissue acquisition, supplemented by endoscopic ultrasound or computed tomography for disease staging. While point-of-care ultrasound (POCUS) is now firmly integrated into clinical practice, it is still not widely utilized. POCUS can be performed at the initial point of contact and provides instant information that can influence investigation strategies. We report two cases of gastric antral carcinoma detected by POCUS, which led to targeted investigations. Both patients underwent expedited upper gastrointestinal endoscopies that confirmed distal gastric carcinoma. These cases highlight the important role of POCUS in triaging patients for timely and appropriate targeted organ investigations.

## Introduction

Gastric cancer is the fifth most common cancer and the third leading cause of cancer death globally [1]. Diagnosis is typically made through endoscopy; however, increasing demand for healthcare services due to a growing patient population may lead to delays in investigation. Point-of-care ultrasound (POCUS) is an important modality that is now firmly integrated into clinical practice, although it remains primarily utilized in certain specialties [2-4]. This is particularly evident in specialties such as the emergency department, where the focused assessment with sonography for trauma (FAST) scan protocol is employed for trauma patients [2], and the bedside lung ultrasound in emergency (BLUE) protocol is applied for acutely dyspneic patients in emergency or critical care settings [3]. POCUS is equally valuable in non-emergency settings, where it can be rapidly performed during initial encounters, guiding further decision-making and directing appropriate targeted investigations. However, the use of POCUS in the assessment of the luminal gastrointestinal tract remains limited. Currently, POCUS or ultrasound for evaluating the stomach is primarily used to assess gastric content and evaluate aspiration risk before anesthesia [4,5].

We report two cases of gastric antral carcinoma detected by POCUS, highlighting its important potential role in evaluating patients presenting with gastric complaints.

## Case presentation

Case 1

A 61-year-old male with a history of diabetes mellitus (HbA1c 6.8%), dyslipidemia, and hypertension presented with a three-day history of persistent dull epigastric discomfort, which was worst on an empty stomach. This discomfort had been mild and intermittent for the past year, controlled with famotidine 20 mg twice daily. On direct inquiry, he reported some weight loss and mild lethargy but denied any altered bowel habits, as well as the passage of altered stool or blood. There was no history of smoking, alcohol use, or family history of cancer. Physical examination was unremarkable. Blood investigations revealed severe iron deficiency anemia (Table 1), characterized by low hemoglobin, low serum ferritin, and a low iron/transferrin ratio

**Table 1:** Laboratory results of Case 1 at admission.

Variables (unit)	Results	Reference range
Hemoglobin (g/dL)	5.3	13.5 - 17.9
Mean corpuscular volume (fL)	67.1	81.0 - 95.4
White cell count (x 10^3^)	11	4.2 - 12.6
Platelets (x 10^3^)	479	174 - 430
Serum iron (umol/L)	4.3	5.8 - 34.5
Transferrin (g/L)	2.99	2.00 - 3.60
Iron/transferrin ratio (%)	5	25 - 45
Ferritin (ng/mL)	6	30 - 400
Sodium (mmol/L)	129	136 - 145
Potassium (mmol/L)	4.0	3.5 - 5.1
Creatinine (mmol/L)	79.8	62.0 - 108.0
Urea (mmol/L)	3.4	2.8 - 8.1
Bilirubin (mmol/L)	7.8	0.0 - 21.0
Amino alanine transferase (U/L)	9	10 - 50
Alkaline phosphatase (U/L)	64	40 - 129
Gamma glutamyl transferase (U/L)	10	10 - 71
Albumin (g/L)	38	35 - 52
Total protein (g/L)	65	66 - 87

**Figure 1:**
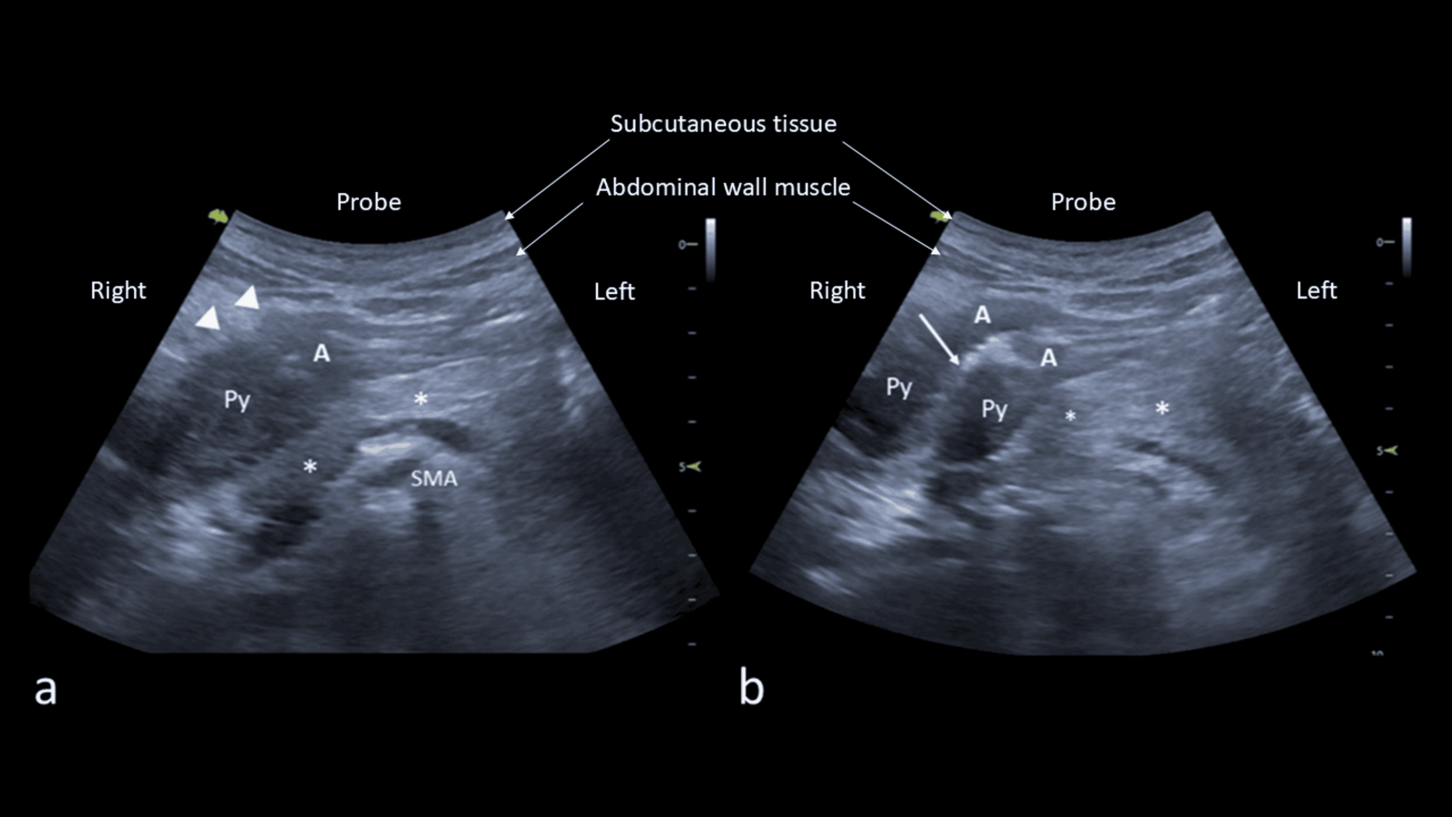
Transverse POCUS images (4.5 MHz) of the epigastrium; a) showing thickened gastric antrum (A) due to the tumor extending to the pylorus (Py) with outer border having an irregular margin indicated by arrowheads located anterior to the pancreas (*), and b) showing the tumor affecting the antrum and pylorus. POCUS: point-of-care ultrasound, A: antrum, Py: pylorus, SMA: superior mesenteric artery.

A chest radiograph showed fibro-bronchiectatic changes in the right upper zone, consistent with old infectious processes, likely from previous pulmonary tuberculosis. In light of the iron deficiency anemia and weight loss, bidirectional upper and lower gastrointestinal endoscopies were planned. A bedside POCUS was performed using a curvilinear probe (4.5 MHz), which identified a thickened antrum with an irregular outer wall (Figure 1). This abnormality extended into the pylorus. There was no evidence of ascites or liver metastases.

**Figure 2:**
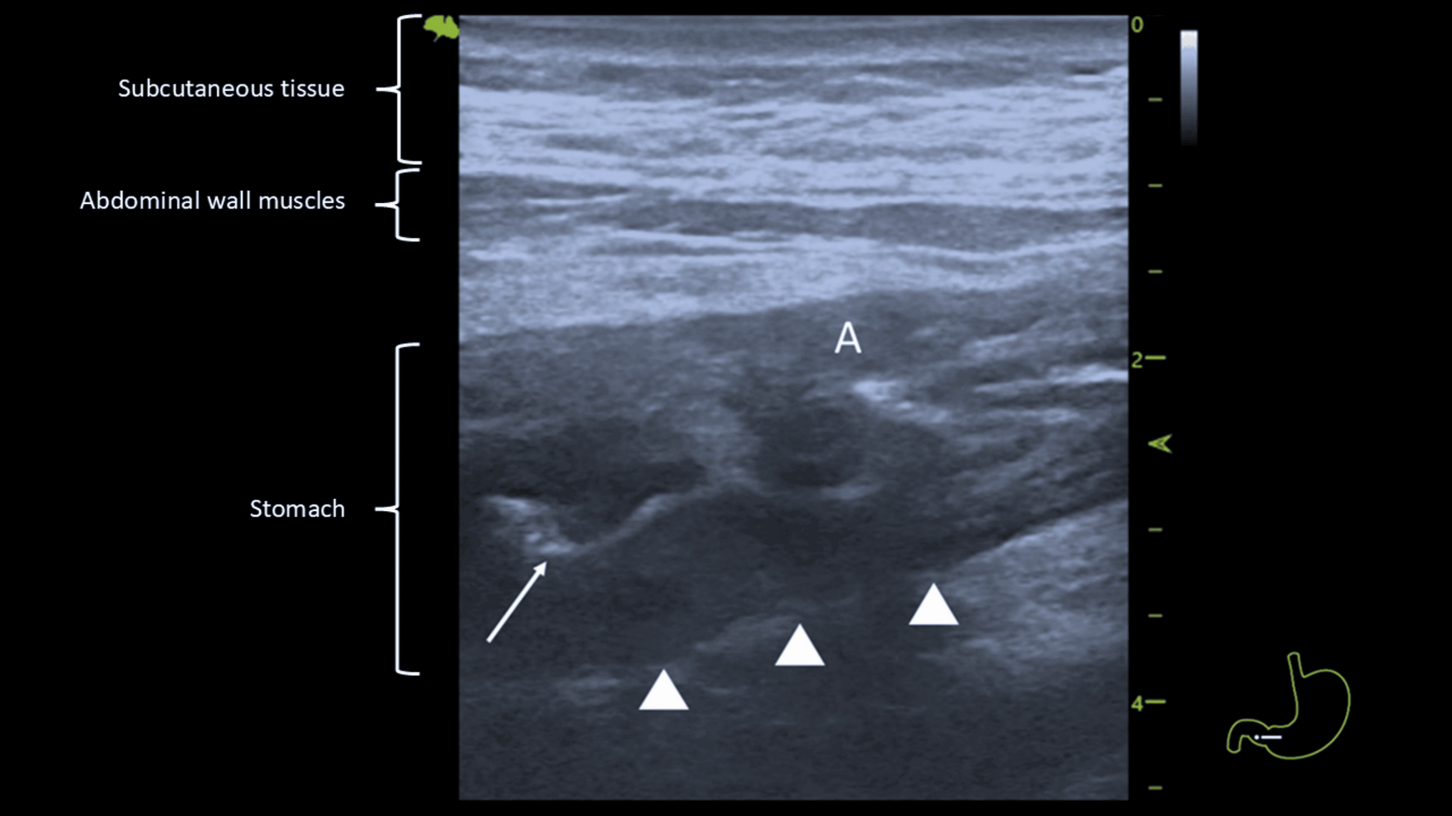
POCUS with a linear probe (8.5 MHz) showing mucosal layer with irregular posterior outer border (arrowheads) and compromised lumen (white arrow). POCUS: point-of-care ultrasound, A: antrum.

A linear probe (8.5 MHz) examination provided a more detailed view of the thickened wall (Figure 2). The POCUS evaluation of the colon identified only normal dirty haustral shadowing.

**Figure 3:**
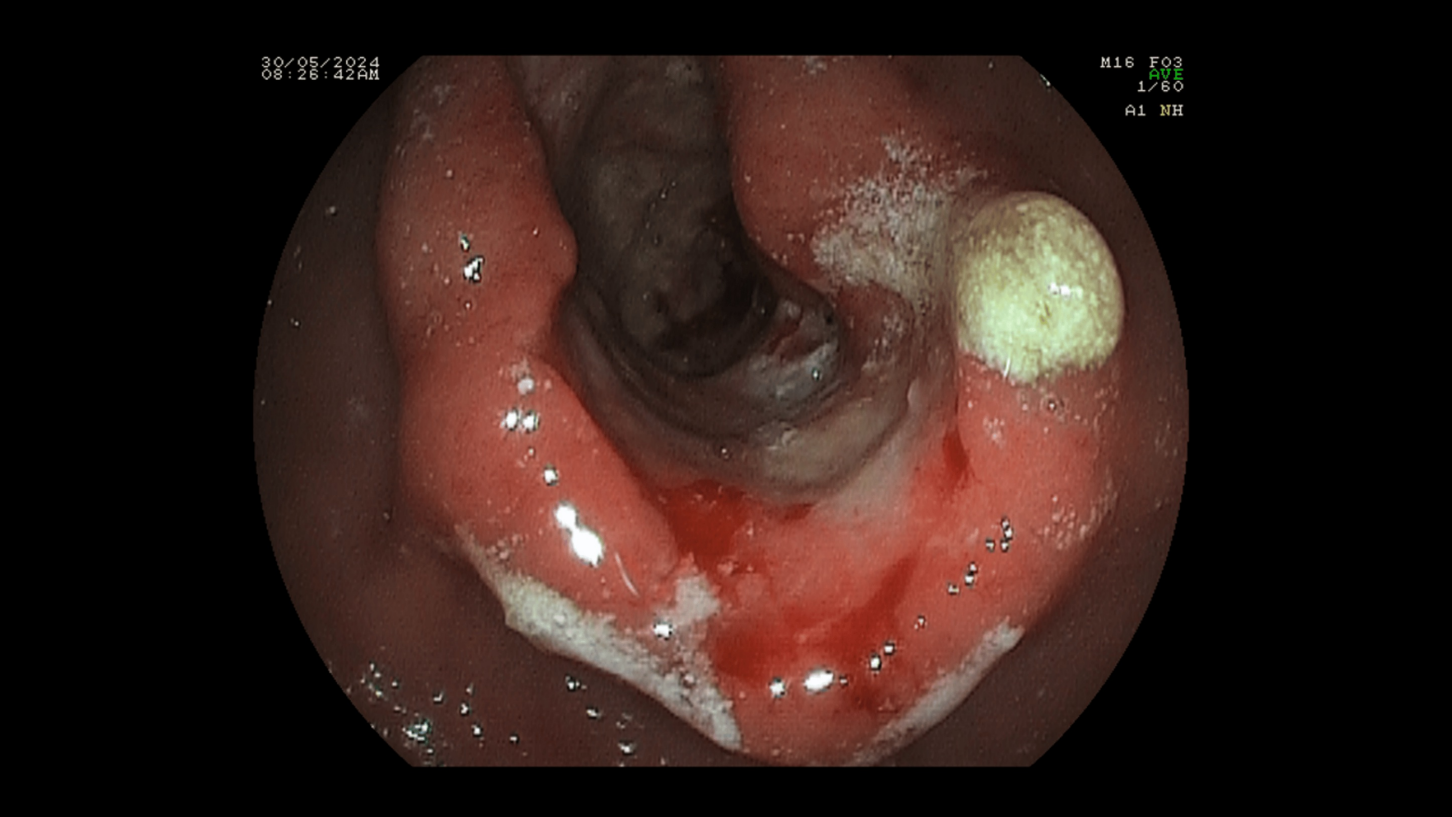
Endoscopic image showing the stenosing gastric tumor located at the antrum.

In light of the POCUS findings, an upper gastrointestinal endoscopy was conducted the following day instead of the planned bidirectional endoscopies. The upper gastrointestinal endoscopy revealed a stenotic tumor affecting the antrum of the stomach (Figure 3), extending into the pylorus and duodenum, consistent with the POCUS findings.

**Figure 4:**
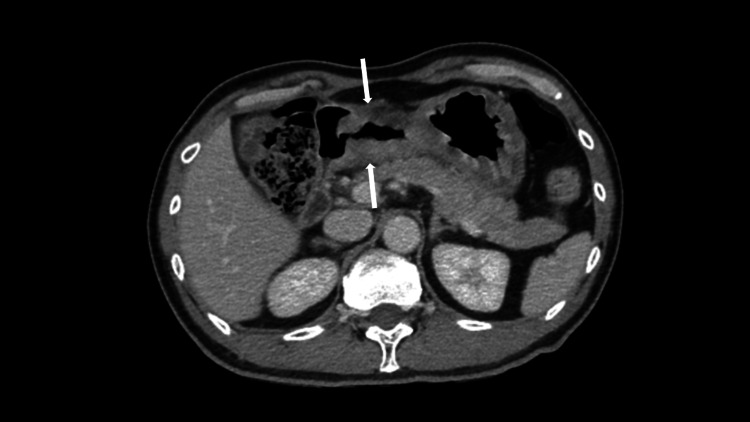
Contrast enhanced CT scan showing thickened gastric antrum anterior and posterior walls (arrows). CT: Computed tomography

Gastric biopsies confirmed moderately differentiated adenocarcinoma. A staging computed tomography (CT) scan of the thorax, abdomen, and pelvis revealed the gastric antral tumor. It showed a peri-gastric lymph node with no evidence of liver or distant metastases (Figure 4).

The patient was referred to the surgical team and underwent a partial gastrectomy. Histology of the resected specimen confirmed moderately differentiated adenocarcinoma staged as pT4a pN3a and tested positive for *Helicobacter pylori infection*. He received eradication therapy and was referred to the National Cancer Centre for chemotherapy. However, a PET scan a month later showed the presence of pyloric lymphadenopathy and mesenteric deposits, indicating aggressive disease. The patient continued with palliative chemotherapy.

Case 2

An 88-year-old man with a medical history of hypertension, gout, bronchial asthma, and chronic kidney disease was admitted due to reduced intake, without any gastrointestinal complaints. On clinical examination, he was afebrile and comfortable, with only mild pallor noted. Physical examinations of the cardiovascular system, lungs, and abdomen were unremarkable. A per rectal examination revealed melena. Blood investigations showed microcytic anemia, with a hemoglobin level of 6.8 g/dL, low serum ferritin and iron, and elevated brain natriuretic peptide (BNP). The results of the other blood investigations were normal (Table 2). Liver function tests conducted two weeks prior to this admission were also normal.

**Table 2:** Laboratory results of Case 2 at and before admission (* done two weeks prior).

Variables (unit)	Results	Reference range
Hemoglobin (g/dL)	6.2	13.5 - 17.9
Mean corpuscular volume (fL)	68.6	81.0 - 95.4
White cell count (x 10^3^)	5.7	4.2 - 12.6
Platelets (x 10^3^)	303	174 - 430
Serum iron (umol/L)	4.2	5.8 - 34.5
Transferrin (g/L)	1.99	2.00 - 3.60
Iron/transferrin ratio (%)	8	25 - 45
Ferritin (ng/mL)	10	30 - 400
Sodium (mmol/L)	135	136 - 145
Potassium (mmol/L)	4.1	3.5 - 5.1
Creatinine (mmol/L)	105.0	62.0 - 108.0
Urea (mmol/L)	6.1	2.8 - 8.1
Brain natriuretic peptide (pg/mL)	2,651	0 - 449
Bilirubin (mmol/L) *	7.6	0.0 - 21.0
Amino alanine transferase (U/L) *	7	10 - 50
Alkaline phosphatase (U/L) *	109	40 - 129
Gamma glutamyl transferase (U/L) *	25	10 - 71
Albumin (g/L) *	33	35 - 52
Total protein (g/L) *	87	66 - 87

**Figure 5:**
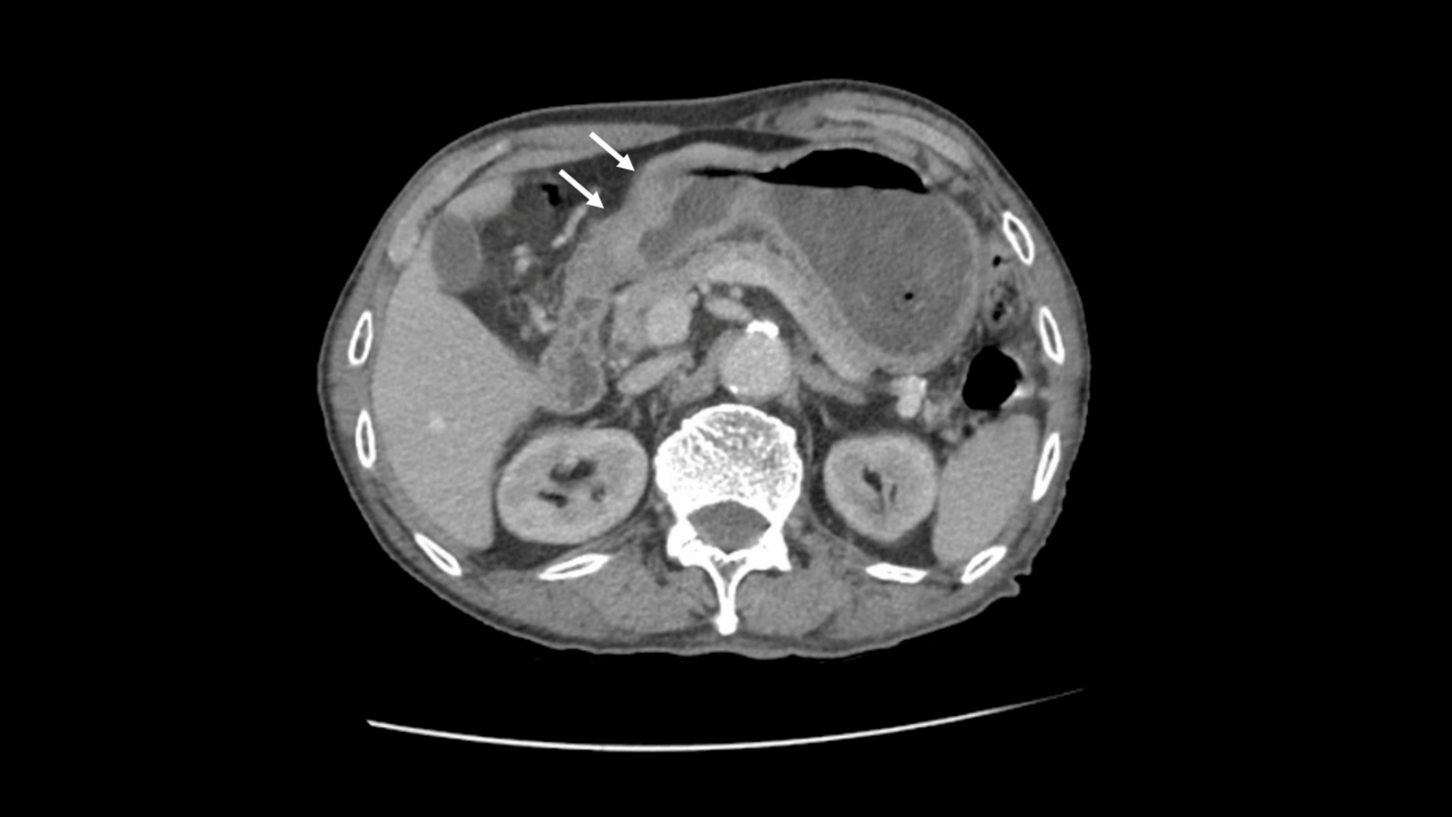
An contrast enhanced axial CT images showing thickened antrum affecting the anterior wall (arrows). CT: computed tomography

He was started on intravenous omeprazole 40 mg twice daily and received one unit of blood transfusion, which raised his hemoglobin to 8.3 g/dL. A bedside POCUS examination of the abdomen with a handheld machine showed a slightly thickened gastric antral wall, which was noted in only one view. The patient was offered an endoscopic evaluation; however, he was not keen on this option. Instead, we proceeded with a CT scan, which revealed a thickened antrum suspicious for gastric carcinoma (Figure 5) but no evidence of distant metastases.

After further discussion, the patient agreed to undergo an upper gastrointestinal endoscopy, which revealed an ulcerated tumor in the antrum affecting less than half of the gastric circumference. Biopsies showed a moderately differentiated adenocarcinoma and were also positive for *Helicobacter pylori*. In view of the age and frail condition, further intervention was not offered, and the patient was managed conservatively. Eradication therapy for *Helicobacter pylori* was also not offered.

**Figure 6:**
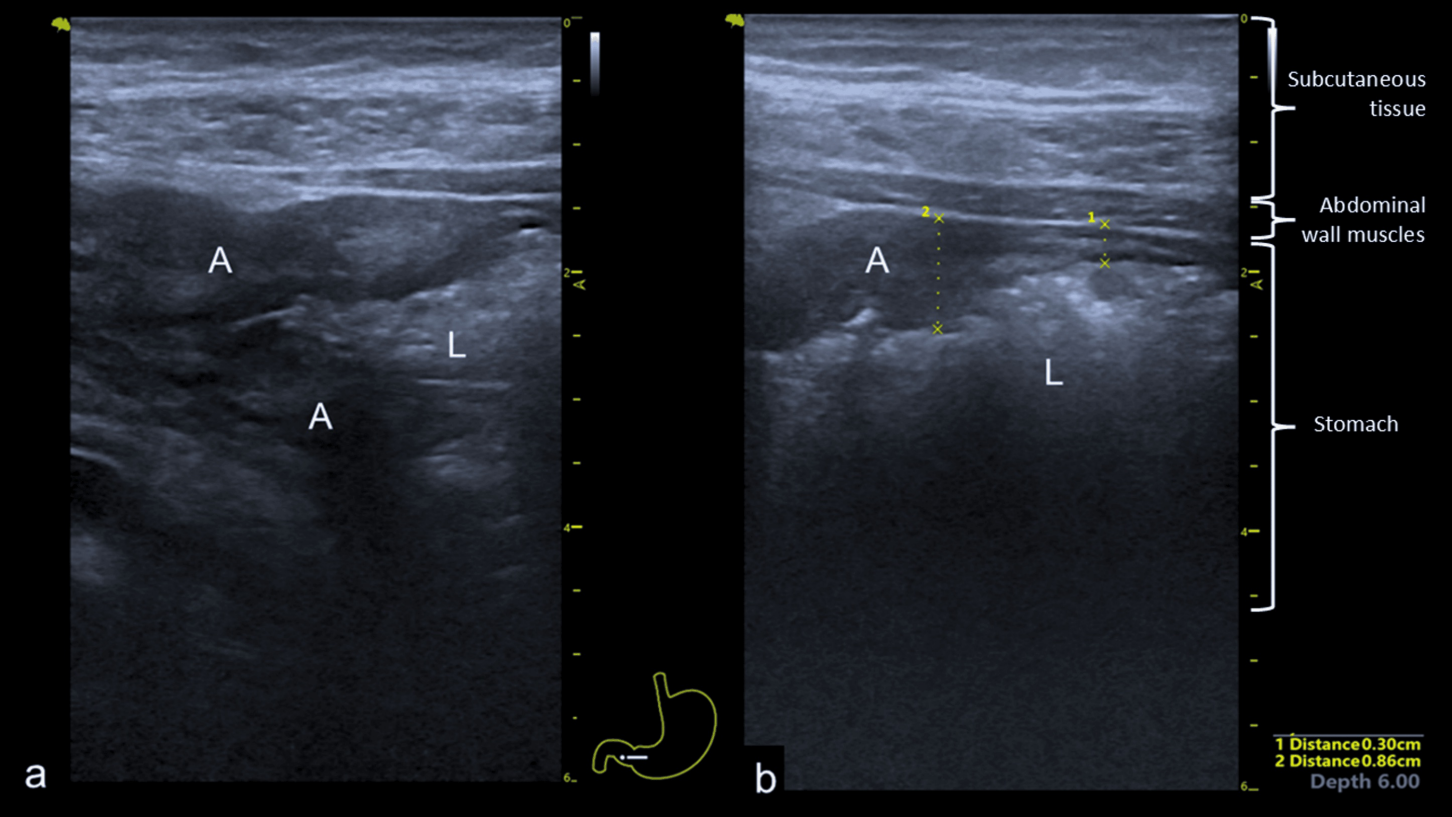
Bedside POCUS showing thickened antrum - a) distal antrum (A) with narrowing of the gastric lumen (L) and b) more proximal antrum without lumen compromise. There is varying wall thickness measuring 8.6 mm at the tumor area and 3.0 mm at the normal part of the stomach. POCUS: point-of-care ultrasound, A: antrum, L: gastric lumen.

He was readmitted four months later with symptomatic anemia and heart failure, denying any upper gastrointestinal symptoms. He was treated for heart failure and received a blood transfusion. A bedside POCUS scan with a dedicated ultrasound machine showed that the tumor had progressed, revealing a thickened antrum affecting both the anterior and posterior walls (Figure 6). He was discharged to palliative care.

## Discussion

We report two cases of gastric antrum carcinomas in which POCUS played a crucial role in detection and management. Both patients were admitted with symptomatic iron deficiency anemia. Case 1 presented with lethargy and worsening dyspepsia, while Case 2 presented with heart failure and gastrointestinal bleeding. In both cases, POCUS indicated the presence of thickened gastric antral walls, leading to expedited and targeted investigations. Although the POCUS abnormality was not as evident at the initial presentation in Case 2, wall thickness and margin irregularities became more apparent in subsequent evaluations after several months of disease progression. These two cases highlight the potential roles of POCUS in detecting and monitoring disease progression.

In clinical practice, patients presenting with various illnesses are often evaluated according to established institutional protocols to avoid delays in diagnosis. For those presenting with iron deficiency anemia, gastrointestinal evaluation is recommended, with bidirectional gastrointestinal endoscopies being preferred [6,7]. These procedures are often scheduled simultaneously, as this approach can potentially shorten investigation intervals compared to a stepwise method. In Case 1, a systematic approach with bidirectional endoscopies was initially planned, following our usual practice. Similarly, in Case 2, although the POCUS findings were not definitive, they still influenced the decision to proceed and directed further investigations. The patient agreed to proceed with endoscopy only after a CT scan confirmed the presence of gastric abnormalities. In both cases, targeted upper gastrointestinal endoscopies were performed following POCUS, demonstrating its dual role in diagnosis and triaging for patients with gastric complaints. This is particularly relevant given the increasing demand for healthcare services due to a growing patient population, which often leads to longer waiting times [8].

Due to the location of the stomach, ultrasound can effectively assess the distal, specifically the distal body, antrum, and pylorus [9-11]. Visualization can be achieved using either a curvilinear or linear probe, particularly for thinner patients. The probe should be placed transversely 1-2 cm below the xiphoid process, allowing for visualization of the pancreas. It can then be moved upward, downward, and laterally to the right to assess the antrum, pylorus, and duodenum. The antrum can also be examined by positioning the probe vertically over the midline, with the superior mesenteric artery, pancreas, and aorta in view. A normal stomach wall consists of five layers: the interphase between the lumen and mucosa, mucosa, submucosa, muscularis propria, and serosa. The normal gastric wall thickness is typically less than 4-5 mm, although it may be thicker towards the pylorus [9,12,13].

One study reported gastric wall thicknesses of 4.9±1.6 mm for patients with benign disease, 5.6±2.4 mm for those with early gastric cancer, and 10.3±4.7 mm for advanced gastric cancer (p<0.01) [12]. A gastric wall thickness greater than 7 mm demonstrated a sensitivity of 75.0%, a specificity of 92.6%, a positive predictive value of 50.0%, and a negative predictive value of 97.4% for advanced gastric cancer [12]. Findings of wall thickness greater than normal, with or without irregular contours or borders, should raise suspicion of underlying neoplasms and prompt further investigation. Gastric cancer typically arises from the mucosal layer, affecting the second hypoechoic layer, which becomes thickened and irregular as the disease progresses, leading to distortion of the other layers. An irregular hyperechoic lumen, especially with proximal distension, indicates lumen compromise. Gastric cancers exhibit distinct features on ultrasound, including thickened walls with margin irregularities [12]. With advancements in technology, imaging now provides superior resolution of the different layers, particularly evident with endoscopic ultrasound [14].

To date, the use of ultrasound or POCUS to assess the stomach has not been extensively researched. The most common clinical application of POCUS is assessing gastric content and aspiration risk prior to general anesthesia [4,5]. Several reports have addressed the role of ultrasound and POCUS in gastric cancer [9, 15-17]. A systematic review indicated that both contrast-enhanced ultrasound (O-CEGUS) and double contrast-enhanced ultrasound (D-CEGUS) can differentiate ≤T1 gastric cancer from ≥T2 gastric cancer, thereby assisting in the formulation of clinical treatment strategies for patients with very early gastric cancer. Given its simplicity and cost-effectiveness, O-CEGUS is often preferred as a staging method for gastric cancer prior to endoscopic intervention [15]. Another meta-analysis found that transabdominal ultrasound (TAUS) is more accurate and sensitive in diagnosing advanced gastric cancer compared to early gastric cancer, although additional features of advanced gastric cancer are needed to enhance recognition. Nonetheless, TAUS can be considered a complementary imaging diagnostic tool alongside CT and endoscopic ultrasound (EUS) [16]. It is important to note that higher-end ultrasound machines, whether dedicated or handheld, yield superior imaging, which is crucial for detecting and characterizing any abnormalities.

POCUS represents a significant advancement in clinical practice, providing rapid, real-time information. Since incorporating POCUS into my daily practice, I routinely use it to assess patients, guide subsequent investigations, and determine their urgency [18]. For patients presenting with warning symptoms such as weight loss, anemia (especially iron deficiency anemia), or altered bowel habits, POCUS can help decide whether targeted or bidirectional endoscopies should be performed. The two cases clearly highlighted this role. Arranging targeted investigations, such as upper gastrointestinal endoscopy, is often easier compared to organizing bidirectional endoscopies due to the availability of endoscopy slots. Additionally, POCUS is valuable for assessing other disorders, including hepatic conditions like pyogenic liver abscesses [18].

## Conclusions

In conclusion, these two cases of gastric antral carcinomas highlight the potential roles of ultrasound and POCUS in evaluating patients presenting with dyspepsia and iron deficiency anemia. Assessment can be performed using either curvilinear or linear probes, which allows for the delineation of the different layers of the stomach. Any abnormal wall thickness, particularly with irregularities in the outer wall margin, should raise suspicion of an underlying neoplasm. Furthermore, positive POCUS findings that localize the site of pathology can help direct the appropriate line of investigation, rather than relying on the shotgun approach commonly used in clinical practice.
